# Aerosol-assisted chemical vapour deposition of transparent superhydrophobic film by using mixed functional alkoxysilanes

**DOI:** 10.1038/s41598-019-43386-1

**Published:** 2019-05-17

**Authors:** Alessia Tombesi, Shuhui Li, Sanjayan Sathasivam, Kristopher Page, Frances L. Heale, Claudio Pettinari, Claire J. Carmalt, Ivan P. Parkin

**Affiliations:** 1 School of Science and Technology, Chemistry Division, University of Camerino, via S. Agostino 1, Camerino, MC Italy; 2 Department of Chemistry, University College London, 20 Gordon Street, London, WC1H 0AJ United Kingdom; 3 National Engineering Laboratory for Modern Silk, College of Textile and Clothing Engineering, Soochow University, Suzhou, 215123 China

**Keywords:** Surface assembly, Nanoscience and technology

## Abstract

A method for the preparation of transparent superhydrophobic silica coatings on glass substrates via aerosol‐assisted chemical vapour deposition (AACVD) is described. A multi-layer process to produce dual scale silica nanoparticles films, by using different functional alkoxysilanes was investigated. A first layer of 3-methacryloxypropyltrimethoxysilane (MPS) and a second layer of tetraethylorthosilicate (TEOS) were deposited at different temperatures to generate micro and nano particles of silica. Finally, a layer of perfluorooctyltriethoxysilane was deposited on top of the two layers to achieve superhydrophobicity. The transparent superhydrophobic film showed transparency of 90% in the visible light region with a static water contact angle of 165° and a sliding angle lower than 1°. Various durability tests were performed on the transparent superhydrophobic film, showing a constant water repellency after corrosion and organic solvents tests, strong resistance under UV light, and thermal stability up to 400 °C. Sandpaper mechanical robustness durability test showed superhydrophobicity for up to 5 rubbing cycles. In this study, a novel strategy to achieve highly transparent superhydrophobic glass surfaces using AACVD of alkoxysilanes, to produce surfaces with excellent durability is described. This shows great potential to obtain silica superhydrophobic films for large–scale applications.

## Introduction

Over the last few years, the development of superhydrophobic surfaces has generated increasing interest and importance in materials science for applied technologies^1,2^. The growing interest towards superhydrophobic coatings is mainly due to their potential performance in various applications in industrial fields such as aerospace, automotive, construction, medical and optical^3,4^. The non-wetting behaviour of the surfaces is essential for many important properties for instance self-cleaning^5,6^, anti-fogging^7^, oil–water separation^8,9^, anti-icing^10,11^ and anti-corrosion^12^. In addition, some practical applications of superhydrophobic surface require that those surfaces retain good transparency, especially in the fields of solar cells, optical devices and windshields.

In general, when a surface has a contact angle (CA) higher than 150° and a sliding angle (SA) lower than 10°, it can be defined as superhydrophobic^13,14^. Generally increasing the water contact angle results in lower surface wettability. To explain the effect of roughness on the CA of a liquid droplet on a superhydrophobic surface, two models are used: the Wenzel model^15^ and the Cassie and Baxter model^16^. Wenzel’s theory assumes that liquid droplet fully penetrates the interstices on the rough surface. This therefore increases the available surface area that can be wetted by the liquid compared to a smooth surface. On the other hand, in the Cassie and Baxter model, the liquid droplet does not wet the whole surface, but lies on the peaks of the superficial roughness, leaving air trapped between the liquid and the surface. Either way, increasing the irregularity of the surface causes the CA of the liquid to increase. Thereby, a superhydrophobic surface increases its hydrophobicity with surface roughness.

One of the key issues in the fabrication of a superhydrophobic surface is the competition between transparency and roughness. Indeed, roughness is an essential requirement for superhydrophobicity but it frequently causes light scattering, which can lead to opacity of the surface causing a lower transparency^17^. To achieve higher transparency of the superhydrophobic films, surface roughness should be lower than the wavelength of visible light (<400–760 nm)^18^. Moreover, superhydrophobicity is also closely related to micrometer- and nanometer-scale roughness on the surface, which along with a low surface energy material leads to apparent CA >150° and lower sliding angle (SA). Efficiency and effectiveness of dual scale roughness is demonstrated in nature, for example with superhydrophobic Lotus leaves^19,20^.

Surperhydrophobic coatings have been fabricated using various methods including etching techniques^21,22^, sol–gel process^23^, laser-ablation^24^, electrospining^25^, spray-coating processes^26^, dip and spin coating^27,28^, layer-by-layer deposition (LbL)^29^ and chemical vapour deposition^30^. The deposition of superhydrophobic films by using AACVD is currently an area of great interest, as it involves a solution-based process that requires solubility of the precursors, which considerably expands the range of possible precursors^31^. The potential to obtain good quality and uniform films can be achieved by controlling the main parameters, such as deposition temperature, deposition time, gas carrier flow-rate and concentration of precursors solutions. AACVD has showed good results in the preparation of superhydrophobic films by using precursors, which vary widely from each other^32,33^.

**Figure 1:**
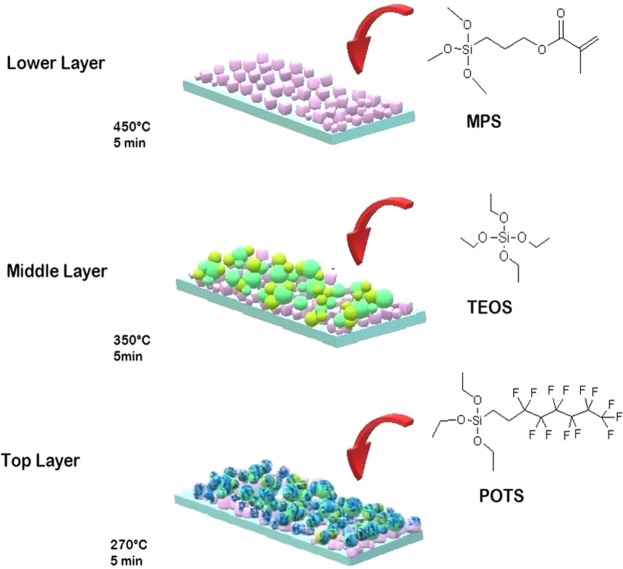
Procedure to fabricate of MPS-TEOS-POTS superhydrophobic film by AACVD.

In the present work, a novel multilayer transparent superhydrophobic coating was fabricated on a glass surface by using functional alkoxysilanes via aerosol assisted chemical vapour deposition (AACVD). It was possible to obtain dual-scale roughness of silica nanoparticle films, through a three-layer deposition of hydrolysed acidic solutions of the following precursors: 3-methacryloxypropyltrimethoxysilane (MPS), tetraethylorthosilicate (TEOS) and 1H, 1H, 2H, 2H perfluorooctyltriethoxysilane (POTS). This multilayer film shows better transparency compared to superhydrophobic films created by AACVD and silica nanoparticles formed by hydrolysis of acidic solutions of TEOS^34^ (Fig. 1). However, these precursors are typically used in the formation of superhydrophobic films by using the sol-gel method followed by deposition techniques such as dip-, spin- and spray-coating^35,36^. Therefore, the most appropriate parameters to obtain an aerosol from a solution of the alkoxysilanes was investigated in detail. The advantages of using three layers was exploited to optimize the water CA, creating dual-scale roughness nanocomposite silica films. In addition, different tests to evaluate the durability in various environments, UV-resistance, mechanical robustness and self-cleaning properties of the surface were studied.

Thanks to the novel strategy adopted in this article, this multilayer silica coating exhibits higher level of transparency and superhydrophobicity by using AACVD compared to existing works. Colin R. *et al*. prepared silica microparticles films deposited using AACVD which showed extreme hydrophobicity, water contact around 180° but films appear white hazy^34^. Zhuang A. *et al*. fabricated a robust superhydrophobic surface depositing epoxy resin and polydimethylsiloxane (PDMS).via AACVD. Such film was found to have transparency in the visible light of 80% but a WCA of 127.4°^37^. Recently, Zhuang A. *et al*. reported preparation of micro/nano- structured transparent superhydrophobic polytetrafluoroethylene (PTFE) films with a water contact angle of 168° and a transmittance to visible light 90% via AACVD^33^.

## Results and Discussion

In the AACVD process, an aerosol is generated from a solution of precursors dissolved in a solvent via the use of an ultrasonic humidifier. The precursor mist is transported by a nitrogen carrier gas to a CVD reactor (cold-wall reactor), where a heated substrate is located. Once inside the reactor, solvent aerosol droplets undergo evaporation; meanwhile precursor droplets can react leading to the formation of a thin film. The morphology and particle size of the deposited film is controlled by the deposition temperature, solvent and deposition time^37^. The precursors solutions SOL-MPS, SOL-TEOS and SOL-POTS were prepared by using acidic aqueous solutions so that alkoxysilane can react with water to form hydrolysed species able to react in the aerosol/reactor to form silica nanoparticles. The hydrolysed siloxanes, by condensation reaction form siloxane bonds (Si–O–Si), and subsequently silica nanoparticles. For MPS, TEOS and POTS, it was necessary to stir the precursor solutions for 1 hour, in order to promote the hydrolysis reaction^38^. The formation of silica nanoparticles was improved by the presence of the acidic catalyst, HCl, and a lower amount of water had to be used for the hydrolysis reaction, in order to obtain a more transparent film (molar ratio SiOR_4_:H_2_O = 1:2). By using a stoichiometric amount of water in the precursor solutions, an opaque film was obtained.

In AACVD, the hydrolysis and condensation reactions take place mainly during the aerosol transport. The aerosol phase of the alkoxysilane precursors, once in the reactor, lose water and solvent via evaporation leaving micro/nano-particles in the gas phase^34^. The precursors react and nucleate in the aerosol to form silicon dioxide particles. These silica particles move away from the immediate vicinity of the heated bottom plate and are attracted towards the colder top plate. This phenomenon, called the thermophoretic effect, allows the deposition of solid particles on the top glass substrate^39^.

Aerosol production efficiency depends on physical parameters related to the liquid nature of the solution^40^. Therefore, it was important to optimize the solution parameters, such as concentration of silicon alkoxide and solvent. The solvents MeOH and EtOH, in a volume ratio 1:1, were chosen for two reasons. First, to have the same carbon chain as the ligands of the alkoxide, to prevent the possibility of ligand exchange. Second, MeOH is more volatile than EtOH improving the formation of the aerosol. Another key factor was the concentration of starting solution. Different concentrations were tested and at lower concentration of precursor solution (0.1–0.4 mol/l) a non-homogenous deposition of the film was obtained caused by the low presence of silica nanoparticles on the substrate (SI Fig. S1a). Higher concentrations (2–2.5 mol/l) resulted in the solution becoming more viscous and unable to be atomized^41^. Moreover, higher concentration of solutions favoured the nucleation of larger and fewer particles with a loss in optical transmission (SI Fig. S1b)^42^. A concentration of 0.9 mol/l and a gas flow rate of 1 l/min were used to deposit homogeneous films. Using higher gas flow rates resulted in less control of the homogeneous and optical quality of the films. The optimum parameters for the deposition of the multilayer films is provided in Table 1.

**Table 1:** Optimum deposition parameters of MPS-TEOS-POTS film.

Deposition parameters	Values
Molar ratio Si(OR)_4_: H_2_O	1:2
R’Si(OR)_3_:H_2_O	1:1.5
Concentration Solution	0.9 mol/L
Solvents MeOH:EtOH	1:1
Carrier gas	N_2_
Gas flow rate	1 L/min

**Figure 2:**
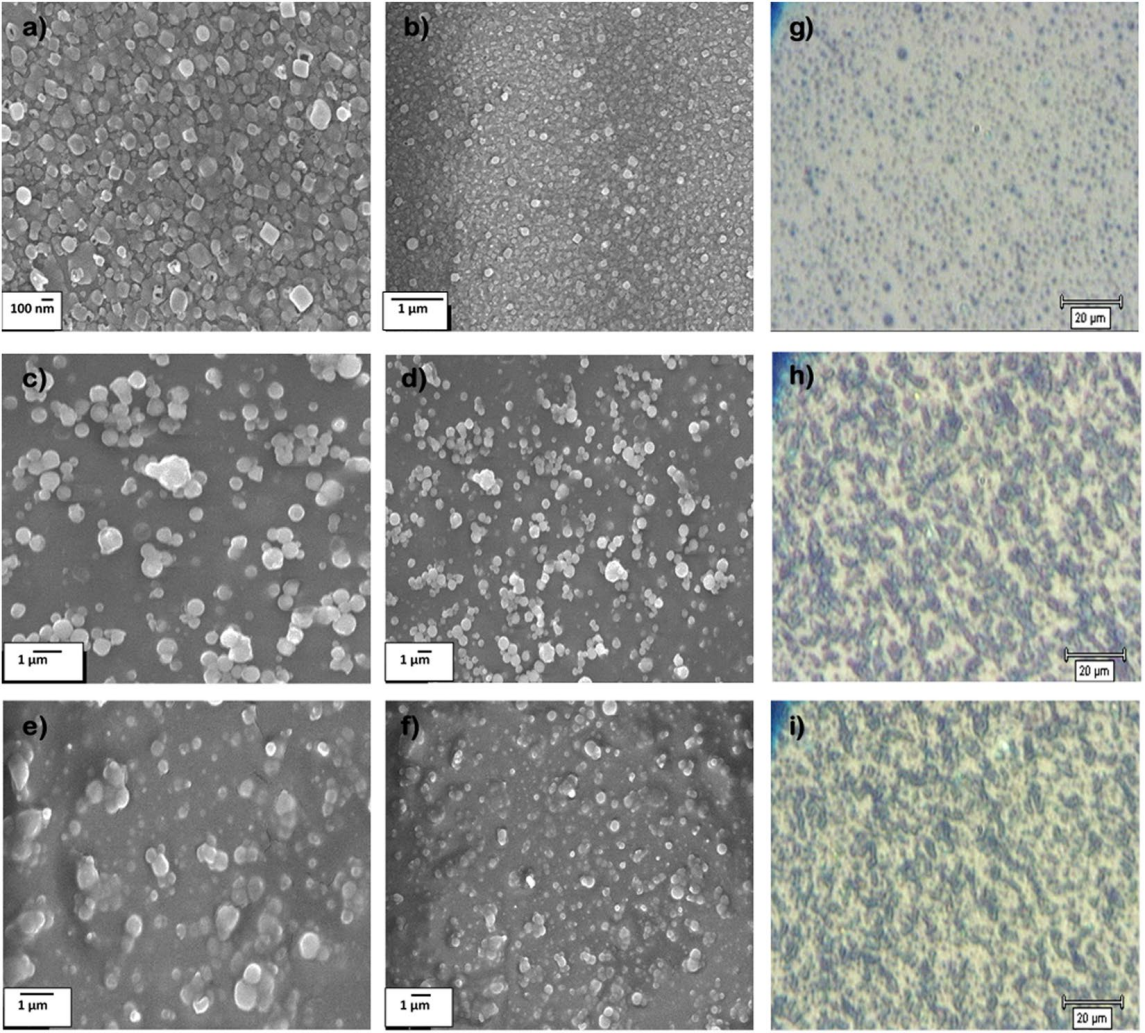
SEM images of the three layer deposited via AACVD. Lower layer deposited at 450 °C at 30000x (**a**) and 20000x (**b**) magnification. Middle layer deposited at 350 °C at 10000x (**c**) and 5000x (**d**) magnification; and Top layer deposited at 270 °C at 10000x (**e**) and 5000x (**f**) magnification. Images from optical microscope 500x of lower (**g**), middle (**h**) and top (**i**) layers Fig. 2 Wettability and contact angle of MPS-TEOS-POTS superhydrophobic film on glass surface.

Colin R. *et al*. showed how different deposition temperatures in AACVD allows for the formation of silica microparticles with various sizes. The silica microparticles surface were converted from superhydrophilic to superhydrophobic with a posttreatment using hexamethyldisilazane (HMDS)^34^. The fabrication process of the MPS-TEOS-POTS film consisted of using different deposition temperatures for each layer, in order to produce a micro and nano-roughness on the surface. The lower layer was formed using MPS nanoparticles deposited at 450 °C via AACVD of SOL-MPS, resulting in the formation of nanoparticles of <100 nm, as shown in Fig. 2a,b,g. The MPS nanoparticles assume a square shape, probably due to the presence of the methacrylate group in the MPS precursor. These nanocube particles, with size range at 20–100 nm, result in almost unaltered glass transparency. Comparison of the glass transparency is provided in SI, after deposition of SOL-MPS (SI Fig. S2a) and SOL-TEOS (SI Fig. S2b) at 450 °C. The observed differences are likely due to changes in particle shape and size at 450 °C. The middle layer consisted of a silica nanoparticles layer deposited from SOL-TEOS at 350 °C. SEM images (Fig. 2c,d) and microscope image (Fig. 2h), clearly show a structure with spherical silica particles arranged in microscopic clusters distributed across the surface. The particle sizes were concentrated in the 300 nm–1 µm range. These aggregates created a number of peaks and cavities thereby roughening the surface. Such structured surfaces has a maximum peak height of 2.17 µm (SI Fig. S3). Finally, the top layer was deposited at 270 °C from SOL-POTS, to lower the surface energy on the silica nanoparticles. Fig. 2e,f,i, shows the morphology after the SOL-POTS deposition. This final layer grafted on the silica nanoparticles through covalent bonds that form via condensation and polymerization reactions between the SiOH of pre-hydrolysed POTS precursor and silanol groups present on the silica nanoparticles^43^.

**Figure 3:**
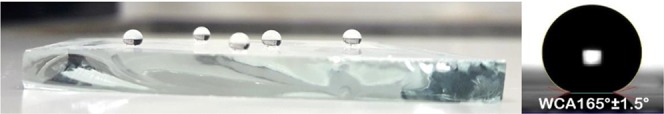
Wettability and contact angle of MPS-TEOS-POTS superhydrophobic film on glass.

In Table 2, the wettability behaviour of the coating made by various combinations of the layers are compared. The lower layer formed by MPS shows a CA around 90°. Then the film fabricated with two layers (MPS-TEOS) showed an increased contact angle of 122° due to micro-nano silica particles. However, with the combination of the full three layers, MPS-TEOS-POTS, a higher water CA, around 165° (Fig. 3), and a lower SA of 0.5° (SI Movie S1) was formed. Therefore, silica nanoparticles modified by the addition of the lower energy compound POTS, greatly increases the hydrophobicity of the layers. The results show a synergetic effect between dual scale micro/nano particles and low surface energy species^44^. The randomly distributed silica particles created by deposition of MPS and TEOS, with a moderately dense coverage of the surface and POTS enables the superhydrophobic properties to be achieved.

**Table 2:** Wettability behaviors of different combination of layers on the glass surface.

Coating composition	Contact Angle [°]	Sliding Angle [°]
MPS-TEOS-POTS	164.9 ± 1.5	0.5 ± 0.1
MPS-POTS	113.6 ± 2.3	82.3 ± 1.1
MPS-TEOS	122.9 ± 1.9	80.5 ± 0.9
TEOS-POTS	139.3 ± 3.2	48.1 ± 1.3
MPS	90.2 ± 4.7	>90

**Figure 4:**
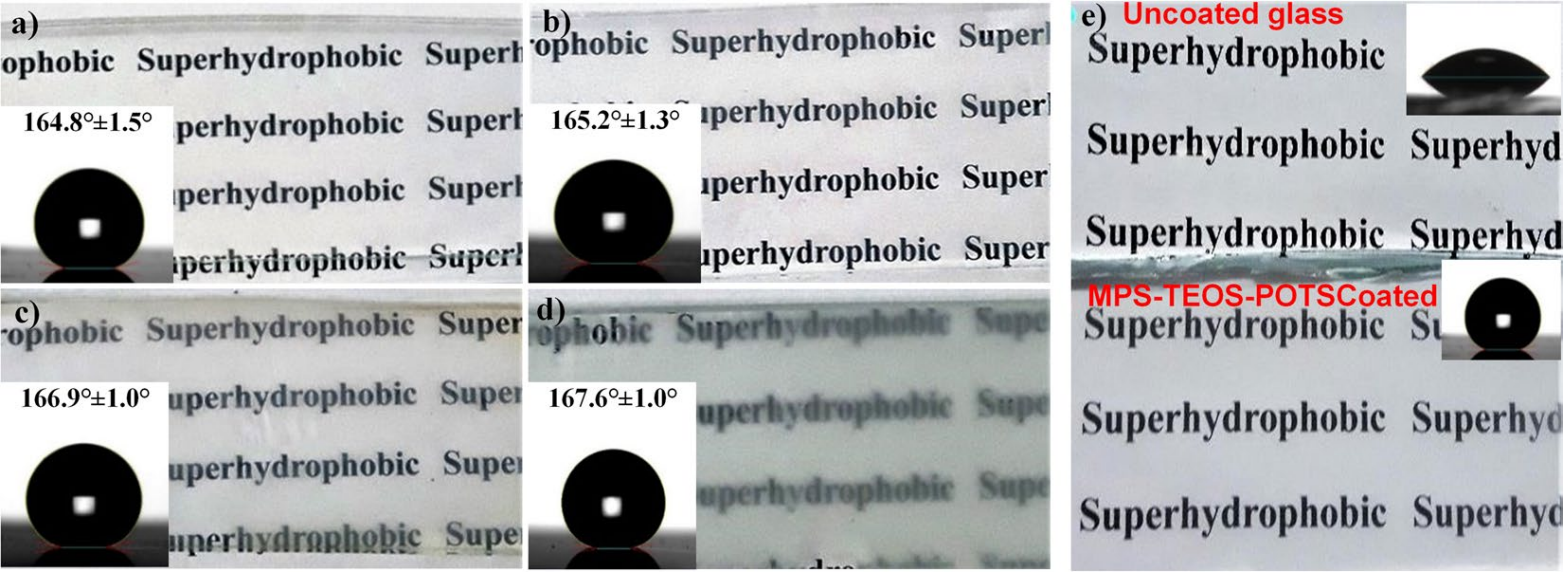
Optical images for visible transparency of MPS-TEOS-POTS film on the glass surface with different deposition time 5 min. (**a**), 6 min (**b**), 7 min (**c**) and 10 min (**d**). In the images (**e**) visual comparison between glass uncoated and glass coated with MPS-TEOS-POTS film.

The relationship between the deposition time of each layer with the transparency and superhydrophobicity was also investigated. The depositions times were increased for each layer of 5 min, 6 min, 7 min and 10 min; keeping fixed depositions temperatures at 450 °C for MPS, 350 °C for TEOS and 270 °C for POTS. Figure 4a–d) shows the visible transparency and CA for the different deposition time. Increasing the time of deposition, resulted in the particle density become greater, thus leading to a higher degree of texture on the surface (SI Fig. S4) and the water CA increase until 169°. In Fig. 4e, the sample at 5 min was compared to an uncoated glass. The sample at 5 min, as shown in Fig. 4a, gives the best transparency while preserving a higher water contact angle.

**Figure 5:**
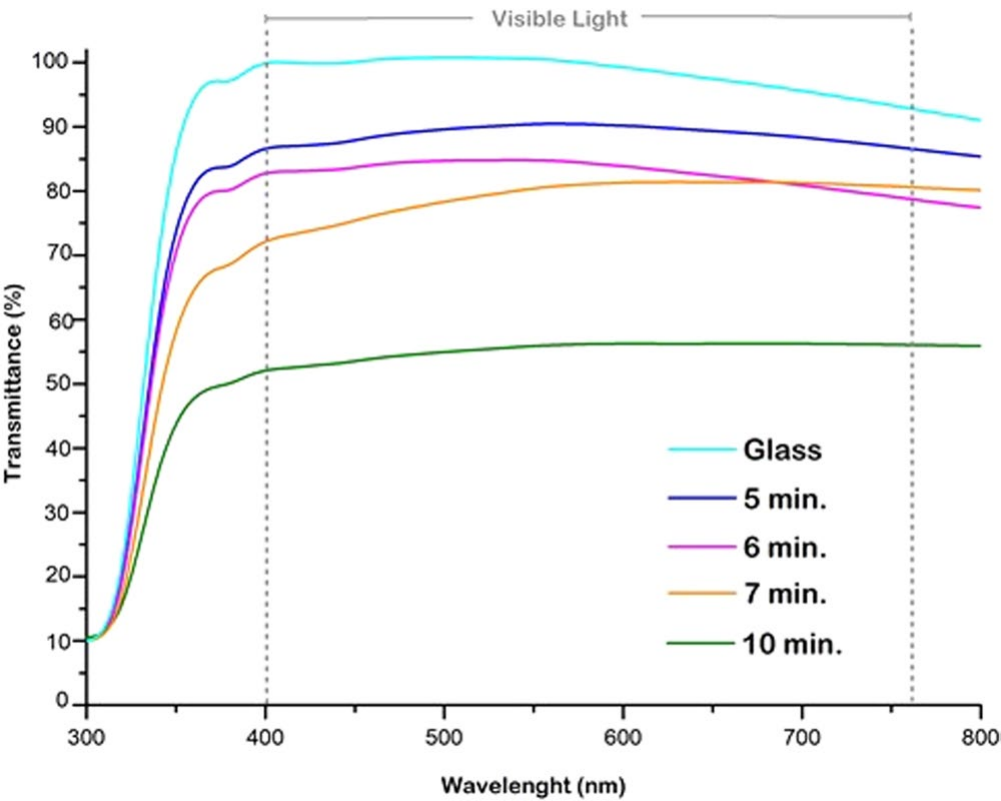
Uv-vis spectra of MPS-TEOS-POTS films deposited at different times with AACVD: 5 minutes (blu line), 6 minutes (pink line), 7 minutes (orange line) and 10 minutes (green line) for each layer.

Optical transmission measurements were performed to evaluate the optical clarity of the coatings obtained at different deposition times. In Fig. 5, the UV-vis spectra of the MPS-TEOS-POTS films at different deposition times were compared with the UV-vis spectra of blank glass. Absorption was examined in the wavelengths 200–800 nm. From UV-vis spectra, in the visible light wavelength range, it was observed that for deposition at 5 minutes (Fig. 5 blue line), the uncoated glass transparency declined by just 10%. In the spectra, it is also possible to see how transparency drastically decreases with deposition time. At a deposition time of 10 minutes (Fig. 5 green line), the transparency was reduced by 50%. In addition, as shown in Fig. 5, there is a constant decrease in transparency on raising the deposition time from 6 (pink line) to 7 minutes (orange line). The deposition at 5 min showed the best transparency property with a water contact angle of 165° and sliding angle lower than 1°, thus it was analysed in more detail.

**Figure 6:**
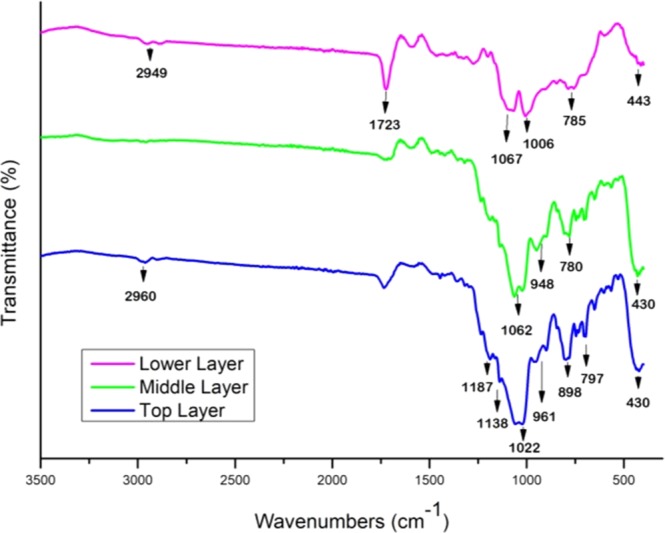
FT-IR spectra of three layer in the MPS-TEOS-POTS coating.

The chemical composition of the MPS-TEOS-POTS coating was identified by FT-IR; EDS and XPS analysis. Figure 6 shows the FT-IR spectra of the lower, middle and top layers within the wavenumber range of 400–3000 cm^−1^. In all FT-IR spectra, the bands from 400 to 1000 cm^−1^ are characteristic for different modes of the Si–O vibrations. The peaks around 430–780 cm^−1^ can be attributed to the rocking transverse and symmetric stretching of Si-O bonds respectively^45,46^. The highest frequency band with the highest intensity at 1000–1070 cm^−1^ corresponds to the asymmetrical stretching of Si-O-Si^47^. In the FTIR spectra of the middle and top layer peaks at 961, 898 and 948 cm^−1^, were observed which are likely due to Si-OH vibrations^48^. The strong absorbance band observed at 1723 cm^−1^ in the lower layer, corresponds to –C=O of the methacrylate group of the MPS^49^. The characteristic peaks at 1187 cm^−1^ and 1138 cm^−1^ are the stretching vibration absorptions of –CF_3_ and CF_2_ group in the top layer. It should be noted that the Si-O peaks in the top layer spectra are slightly broadened and this may be due to partial overlap of the C-F peak with the siloxane peaks^50,51^. In addition, weak peaks at 2950 cm^−1^ caused by the C-H stretch of CH_2_ groups were present in MPS and POTS precursors.

The chemical composition of the film was also confirmed by Energy Dispersive X-Ray Spectroscopy (EDS) microanalysis. The EDS spectra of the three layers confirms a composition consistent of silica nanoparticles, which gave a Si: O ratio of 1:2 in each spectrum. Furthermore, EDS spectra of the lower and top layer shows the presences of C and F, in agreement with the FTIR data spectra.

**Figure 7:**
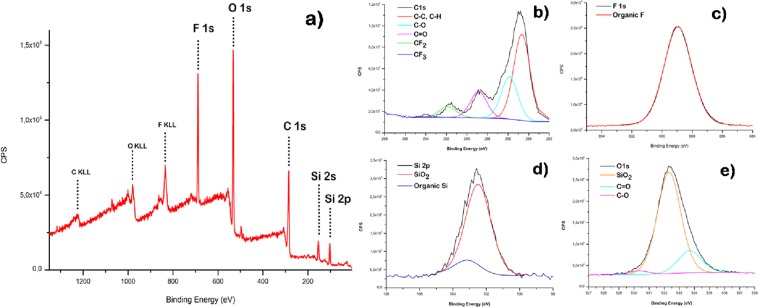
XPS survey spectrum of superhydrophobic film (**a**) and high resolution spectra of C 1s (**b**), F 1s (**c**), Si 2p (**d**) and O1s (**e**).

X-ray photoelectron spectroscopy (XPS) was employed to analyse chemical states and composition of elements present in the film surface, at a sampling depth of 2–10 nm^52^. In the XPS survey spectrum binding energies of 102.5, 284.8, 533.3, and 688.8 eV were observed, indicating the presence of Si, C, O, and F, respectively. Moreover, to further analyse the superhydrophobic film, high-resolution spectra of Si 2p, F 1s, C 1s and O 1s were obtained, as shown in Fig. 7a. The C 1s region of the XPS spectrum (Fig. 7b), showed that the carbon atoms were present in five chemical states. The peak at 284.6 eV corresponds to carbon linked to hydrogen C-H or carbon C-C; at 285.66 eV to carbon linked to one oxygen C-O; at 288.8 eV to one carbonyl oxygen C=O and at 291.8 and 293.8 eV to CF_2_ and CF_3_ bonds of the fluoroalkyl group^53^. Furthermore, Fig. 7c shows the F 1s spectra, where it is evident that the typical symmetric peak of organic fluorine was present at 688.9 eV. The elements that act as a marker for silica nanoparticles are the Si 2p and O 1s signals. The intense peaks due to silica nanoparticles, in the spectra of Si 2p and O 1s (Fig. 7d,e), were at 102.2 and 532.3 respectively^46^. Table 3 provides the atomic percentages for the content of the surface.

**Table 3:** XPS Surface composition of superhydrophobic MPS-TEOS-POTS film.

Components	Position	% Area
**C1s**		
C-C; C-H	284.66	51.22
C-O	285.66	28.66
C=O	288.86	13.08
CF_2_	291.56	6.49
CF_3_	293.86	0.24
**O1s**		
SiO_2_	532.33	80.28
C=O	533.70	17.31
C-O	530.33	2.41
**Si2p**		
SiO_2_	102.49	86.76
Organic Si	103.12	13.24
**F1s**		
Organic F	688.95	100

**Figure 8:**
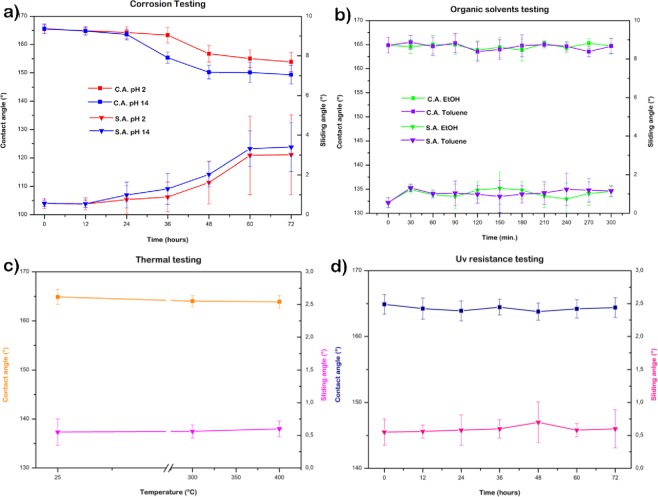
Contact angle and sliding angle of MPS-TEOS-POTS film after corrosion test (**a**), organic solvents test (**b**), thermal test (**c**) and UV resistance test (**d**).

The durability of superhydrophobic coating is an issue that currently represents a hindrance for the technology transfer from laboratory to industrial applications. Therefore, different tests were performed to evaluate the resistance of the MPS-TEOS-POTS film in various environments. Corrosion testing was performed by immersing the films for 72 h in hydrochloric acid (pH = 2) and sodium hydroxide (pH = 14) solutions. The CA and SA were checked every 12 h. The CA and SA of the surface were still unchanged after 48 hours. However, after 48 hours soaking in acidic and alkaline solutions a slight lowering of CA and a raising for SA was observed. This change at 48 h was more evident for the sample in alkaline environment, for CA and SA until 150° and 3°, respectively Fig. 8a, due to the increase in surface hydroxyl groups which are generated by these processes.

Supplementary test was performed to evaluate the behaviour of the MPS-TEOS-POTS film in an organic environment. The surface was tested in two different kind of the solvents, in order to observe the effect of a slightly polar organic solvent, (ethanol EtOH) and of an apolar solvent (toluene). The samples were immersed in organic solvent for 5 hours; CA and SA were checked every 30 min. Organic solvent test results are shown in Fig. 8b. It can be seen that the samples resist both the ethanol test and toluene test, without any change in CA and SA. In Fig. 8c, the heating test results are illustrated, after 5 hours at 300 °C, and then at 400 °C. The contact angle remained high at 165° and sliding angle lower than 1°. Hence, the samples resisted prolonged thermal stress. The UV light resistance test was carried out because UV radiations can be potentially harmful for a material. In order to test the UV resistance of the superhydrophobic multilayer surfaces, the samples were exposed to UV light (365 nm, 3.7 mW/cm2) for 72 h at room temperature, and CAs and SAs of the resulting surfaces were measured after each 24 h period as shown in Fig. 8d. The results showed excellent UV stability no changes on wettability, preserving the CA and SA.

**Figure 9:**
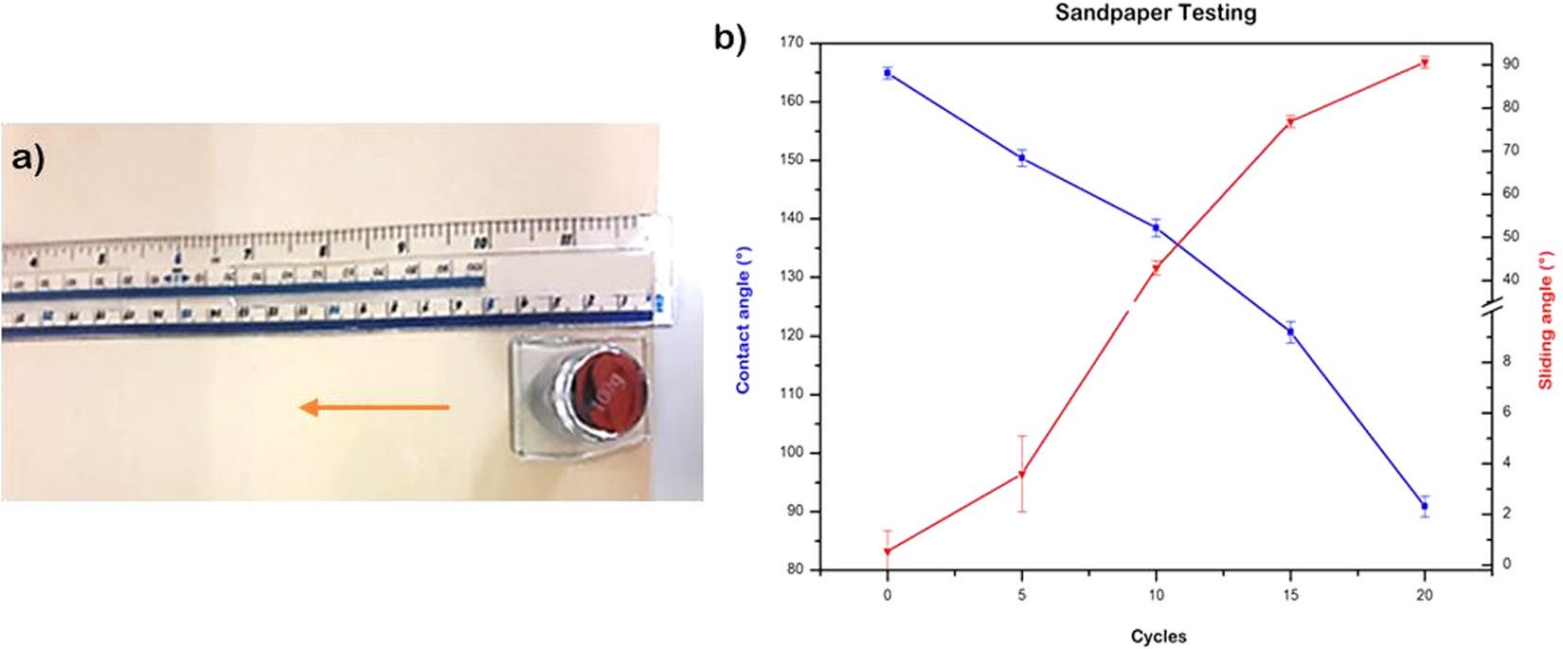
Sandpaper abrasion test. The sample was positioned face-down to sandpaper and moved for 20 cm along the ruler; the sample was rotated by 90° (face to the sandpaper) and then moved for 20 cm along the ruler (**a**). The contact angle and sliding angles of MPS.

Mechanical durability is another important factor that affects the practical application of superhydrophobic materials, especially mechanical friction that leads to the damage of the surface micro/nano structure, resulting in the loss of superhydrophobicity. In this work, a sandpaper abrasion test was used to examine the surface mechanical durability of the MPS-TEOS-POTS film fabricated at 5 minutes. Figure 9a shows detail of how this was carried out, and in Fig. 9b, the contact angles and sliding angles after 5, 10, 15 and 20 of abrasion cycles are shown. At 5 cycles, which is equivalent to 1 m in distance, the surface retained a contact angle of 150° and sliding angle around 3°. However, the CA reduced to 138° after 10 cycles and 120° after 15 cycles (3 m), which is an indication that the top layer was completely removed from the surface (Table 2). Increasing the cycles to 20, i.e. 4 m in distance, the contact angle went down to 90°, corresponding to the lower layer on the surface such that most of the middle layer has been removed. The SEM imagine (SI Fig. S5) showed the MPS-TEOS-POTS film after having been subjected to the abrasion test, the surface micro- and nanostructured has been removed by abrasion-induced. In order to overcome this problem, an adhesive layer between MPS-TEOS-POTS film and substrate could be used. The silica particles could be more stably fixed and linked to the glass substrate throughout a highly adhesive bottom layer. Incorporating new high mechanical stability precursors in coating could be another possible approach to mitigating this effect.

**Figure 10:**
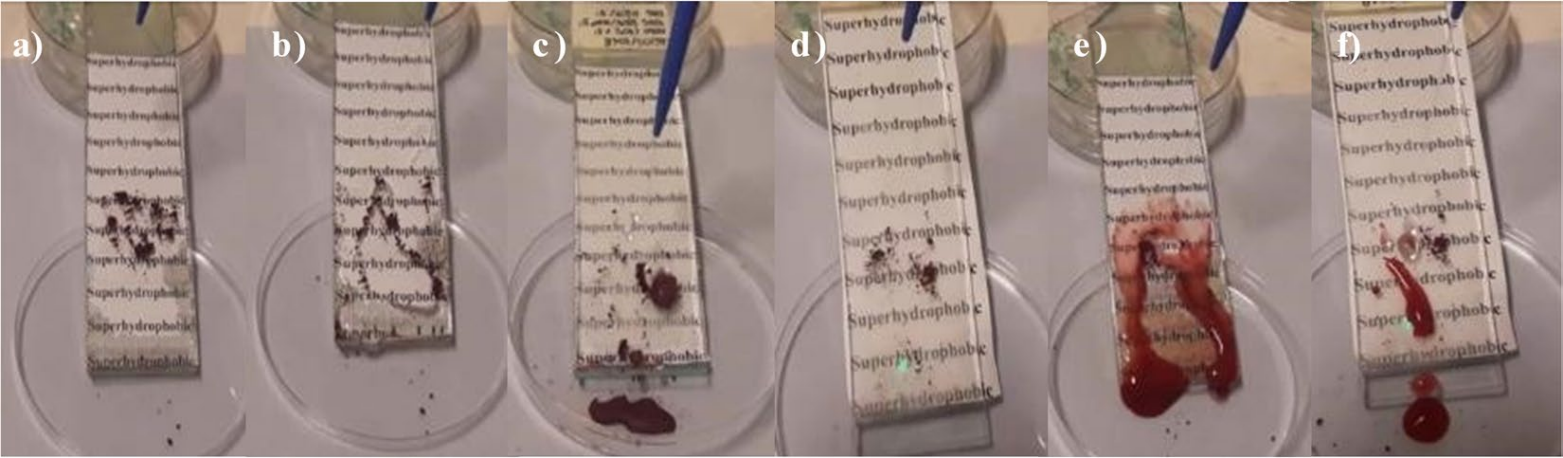
Self-cleaning test. Deposition of contaminates on the surface methyl red (**a**) and congo red (**d**). Self-cleaning process of uncoated glass from methyl red (**b**) and congo red (**e**) and self-cleaning process of glass coated with MPS-TEOS-POTS film from methyl red (**c**) and congo red (**f**).

The self-cleaning property is an important feature in technical applications ranging from self-cleaning window glasses, paints, and textiles and include low-friction surfaces for fluid flow and energy conservation^53^. To demonstrate the self-cleaning performances of MPS-TEOS-POTS coating, Congo red and Methyl red dyes were used as contaminants. The Fig. 10 shows the self-cleaning behavior of uncoated glass and coated glass with MPS-TEOS-POTS film; Methyl red dye as a contaminant insoluble in water in Fig. 10a–c and Congo red as a contaminant soluble in water in Fig. 10d–f. The dye powder was randomly distributed on the two surfaces, and then they were cleaned with a jet of water. Impacting and rolling water drops removed the contaminating particles on the coated surface^54^, it can be seen in the SI Movies S2–5 how all particles were easily removed from the surfaces when subjected to jet of water. The results showed that superhydrophobic coating remains clean without any dust, after the passage of water, compared to uncoated glass.

The coating described in this paper comprises three layers, deposited separately by AACVD which helps the creation of transparent, durable superhydrophobic coating. The lower and middle layers have created a silica nanoparticles dual scale roughness, keeping excellent optical performance. Finally, the fluoro-based top layer provides water-repellency because of its low surface tension chain. It is well known that the coexistence of surface roughness and low surface energy material are requisites for achieving superhydrophobicity^55–57^. Hence, highly transparent superhydrophobic, environmentally durable and self-cleaning MPS-TEOS-POTS coating can be easily applied in industrial applications as architectural glazing, screens for electronic devices and protective layers for solar panels^58–60^.

## Conclusion

Transparent superhydrophobic film with hierarchical micro/nano-structured MPS-TEOS-POTS was prepared on glass substrates using aerosol-assisted chemical vapour deposition. To accomplish this combination of properties, a three layer assembly method was utilized to control the placement and level of aggregation of differently sized nanoparticles within the resultant multilayer thin film. The films demonstrated water droplet contact angles up to 168°, sliding angles less than 1° and excellent optical transmittance exceeding 90% in the visible range (400–760 nm). In order to obtain a micro/nano structure and transparent superhydrophobic surfaces from alkoxysilane precursor by using AACVD, deposition parameters and concentration of the solutions plays an important role.

The coating demonstrated a superhydrophobic performance and excellent stability, after having been subjected to durability testing as corrosion, UV-resistance, organic solvents and thermal tests.

The mechanical robustness testing indicated that the film retains superhydrophobicity after 5 cycles (1 m) after abrasion test. Meanwhile, the film also demonstrated excellent performance in self-cleaning testing. The procedure we have described ensures that the transparent superhydrophobic coating results from alkoxysilane *via *AACVD. The possibility to deposit such films would increase again the potential of this technique and provides a new potential route for the preparation of higher transparent silica superhydrophobic coating in large –scale applications.

## Methods

### Materials

Tetraethylorthosilicate 98% (TEOS) and 3-methacryloxypropyltrimethoxysilane 98% (MPS) were purchased from SigmaAldrich U.K., 1H, 1H, 2H, 2H Perfluorooctyltriethoxysilane 97% (POTS) was purchased from Alfa Aesar. Hydrochloric acid (HCl 37%) was purchased from Sigma Aldrich U.K. Methanol and absolute ethanol were purchased from Fischer Scientific, U.K. The SiO2 barrier glass (dimensions: 145 × 45 × 5 mm) was supplied by Pilkington NSG Ltd. Micrograined sandpaper (TUFBAK, ADALOX, P1000) was bought from Norton Company. Congo red and Methyl red dyes were supplied by BDH Chemicals Ltd.

### Precursors solutions

Precursors solutions were prepared, one for each precursor. The solutions were prepared by mixing alkoxysilane and deionized water with HCl as catalyst, absolute ethanol and methanol as solvent in a volume ratio 1:1. All precursors solutions were prepared with an alkoxysilane concentration of 0.9 M in absolute ethanol-methanol mixtures. Solution of 3methacryloxypropyltrimethoxysilane (SOL-MPS) was prepared in a molar ratio 1:1.5 with a aqueous acidic solution of HCl 0.1 M; solution of tetraethylorthosilicate (SOL-TEOS) in a molar ratio 1:2 and solution of 1H, 1H, 2H, 2H perfluorooctyltriethoxysilane (SOL-POTS) in 1:1.5. In all solutions, processing was initiated by mixing the alkoxide and ethanol-methanol for 15 min. In the second step, aqueous HCl solution was added dropwise to the alkoxide/ethanol-methanol solution under constant stirring at room temperature. After stirring for 1 hour under atmospheric conditions, the solutions were used to prepare the films.

### Fabrication of superhydrophobic film

The surface of the glass substrate was cleaned with aqueous solution of NaOH, a pH >9, for 15 minutes, followed by treating with an acidic aqueous solution of HCl 0.1 M for another 15 minutes. Then, the glass was rinsed in deionized water and dried in air.

The deposition process of the materials consisted of coating the glass surface using an AACVD reactor, described previously^61^, and was carried out in a cold-walled horizontal CVD reactor. The CVD reactor consists of a quartz tube that enclosed a carbon block for heating, on which the bottom plate was placed and a top plate was positioned 8 mm above and parallel to the bottom plate. The aerosol was formed using a PIFCOHEALTH ultrasonic humidifier, operating at a frequency of 40 kHz and 25 W of power. A nitrogen gas flow, with a rate at 1 l/min, was used to move the aerosol from a cylindrical mixing chamber to the CVD reactor, and it was continued throughout the deposition time. The superhydrophobic films were deposited using 20 ml of the precursor solutions, previously described in section 2.2, for the three main parts of the film: lower, middle and top layers, as shown in Fig. 1. The multilayer MPS-TEOS-POTS film was created by depositing three layers, with the AACVD reactor, each at a different temperature but with a constant deposition time. First, the lower layer, in contact with the substrate, was obtained by depositing SOL-MPS at 450 °C. Then, the deposition of the middle layer was performed at 350 °C using SOL-TEOS. The top layer was deposited from SOL-POTS at 270 °C. Depositions were carried out for various durations 5 min, 6 min, 7 min and 10 min for each layer. Once all 3 layers were deposited, the film was cooled *in situ* in the reactor under a flow of nitrogen gas, and subsequently handled and stored in air.

### Characterisation

The surface morphologies of the sample were studied using a JEOL JSM-6301F field emission scanning electron microscope (SEM). A thin layer of gold or carbon was sputtered onto the sample surfaces under vacuum to improve the electrical conductivity. Energy dispersion spectroscopy (EDS) was also carried out on the same machine and was used to obtain the chemical composition of the film. An optical Leica DM LM microscope was used for examination of the layers.

Fourier transform infrared spectroscopy (FT-IR) were recorded on a Bruker FT-IR Platinum ATR single reflection, over the range of 400 to 4000 cm^−1^. The samples for FT-IR characterization were prepared by removing the film from the substrate using a stainless steel knife. X-ray photoelectron spectroscopy (XPS) was carried out on a Thermo Scientific K-alpha photoelectron spectrometer with monochromatic Al-Kα source to identify the chemical state and constituents of the film. Ultraviolet/visible (UV/Vis) spectroscopy was conducted using a Shimadzu UV-VIS 2600 spectrophotometer. Water contact angles (CA) were measured with a FTÅ 1000 B Class instrument with a 7 µl water droplet used and the sliding angle (SA) measurement were performed using the tilted drop method with a water droplet volume of 15 µl and were measured with a Silverline digital angle level. It was tested at five different positions on each sample, and the average value was used.

### Durability testing

A range of tests was performed to evaluate the durability and resistance of the multilayer superhydrophobic surface^60^. A set of the tests were carried out including stability in acidic and alkali environment, stability in organic solvent, UV light resistance and heat resistance. The CAs and SAs were measured before and after each test to estimate the eventual decrease of superhydrophobicity.

In the UV resistance test, the samples were exposed to UV light (365 nm, 3.7 mW/cm^2^) for 72 h at room temperature, the wavelength was chosen to be as close as possible to the common external UV irradiation. In order to determine the anticorrosion resistance of the film, the samples were immersed in alkali (NaOH) and acidic (HCl) aqueous solution (pH = 1 and 14) for 72 h, then rinsed until neutral with pure water and dried in air. To observe the effect of slightly polar organic solvent and of an apolar solvent, the surfaces were tested in two different kinds of solvents, ethanol and toluene respectively, for 5 hours. The heat thermal resistance was conducted in an oven, at 300 °C for 5 h and subsequently at 400 °C for a further 5 h to measure the samples resistance to prolonged thermal stress.

### Mechanical robustness testing

The mechanical robustness property of the as-prepared superhydrophobic film was demonstrated by sandpaper (Standard England sandpaper grit no. 240). A 40 × 30 mm sample was placed onto the sandpaper sheet, with the superhydrophobic film side into direct contact with the abrasive sheet. Then, a 100 gr weight was put on the middle of the sample and moved for 20 cm along the ruler. The sample was rotated by 90° (face to the sandpaper) and then moved for 20 cm along the ruler, thus ensuring the longitudinally and transversely abrasion of the surface.

### Self-cleaning testing

The self-cleaning properties were investigated by dispersing dye powders on the film surface, which were removed through water droplets being continuously dropped on the surface. The superhydrophobic glass was supported in a petri dish with a determined tilt angle. The dye powders, Congo red and Methyl red, were used to simulate contaminants or dusts on the surfaces. When the surface remained clear without contaminants, the film shows excellent superhydrophobic properties and low surface energy^61^.

## Supplementary information

SI -Movie S1

SI- Movie S2

SI- Movie S3

SI-Movie S4

SI-Movie S5

Electronic supplementary information
