# Supplementary material for: Aerosol-assisted chemical vapour deposition of transparent superhydrophobic film by using mixed functional alkoxysilanes
Source: Sci Rep. 2019 May 17;9:7549. doi: 10.1038/s41598-019-43386-1 (PMC6525186; doi:10.1038/s41598-019-43386-1)
Supplement: Supplementary file 6 — Electronic supplementary information [file 41598_2019_43386_MOESM6_ESM.docx]

Electronic supplementary information

Aerosol-assisted chemical vapour deposition of transparent superhydrophobic film by using mixed functional alkoxysilanes

Alessia Tombesi^a,b^, Shuhui Li^b,c^, Sanjayan Sathasivam^b^, Kristopher Page^b^, Frances L. Heale^b^, Claudio Pettinari^a*^, Claire J. Carmalt^b^ and Ivan P.Parkin^b*^

^a^*School of Science and Technology – Chemistry Division, University of Camerino, via S. Agostino 1, Camerino, MC, Italy*

^b^*Department of Chemistry, University College London, London WC1H 0AJ, United Kingdom*

^c^*National Engineering Laboratory for Modern Silk,College of Textile and Clothing Engineering, Soochow University, Suzhou 215123, China*

***Corresponding author, E-mail: [i.p.parkin@ucl.ac.uk](mailto:i.p.parkin@ucl.ac.uk); E-mail: [claudio.pettinari@unicam.it](mailto:claudio.pettinari@unicam.it)

**
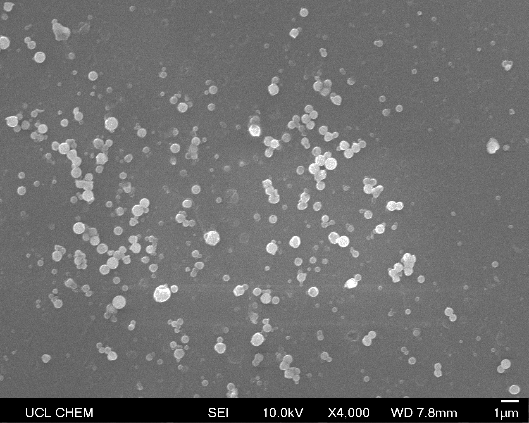

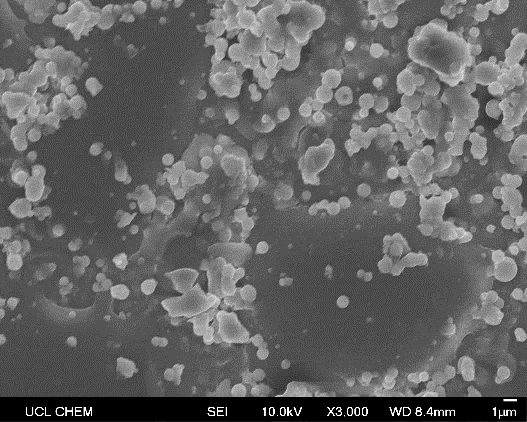
**

**Figure S1** SEM imagine of MPS-TEOS-FAS coating at lower concentration solutions 0.2 mol/l **a)** and higher concentration solution 2.5 mol/l **b)**

a)


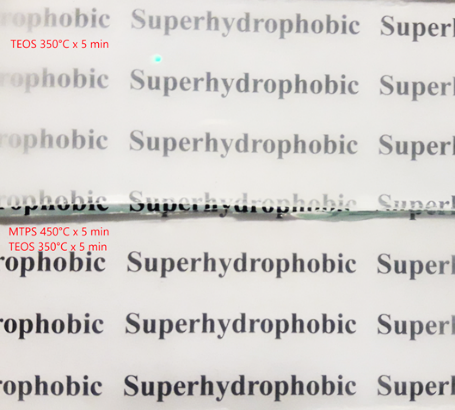

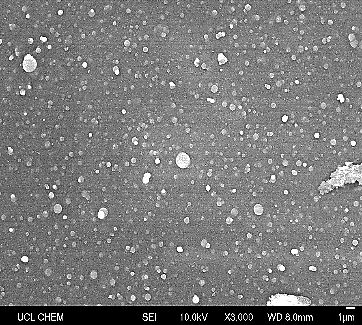

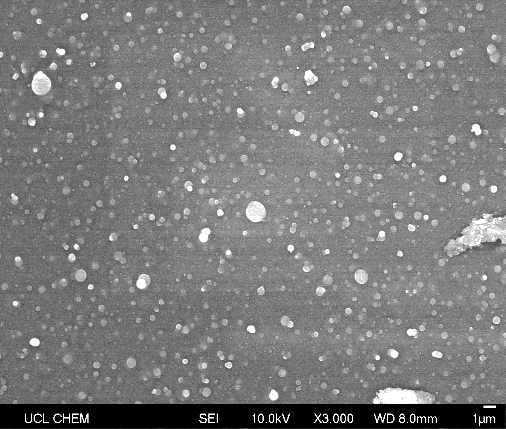

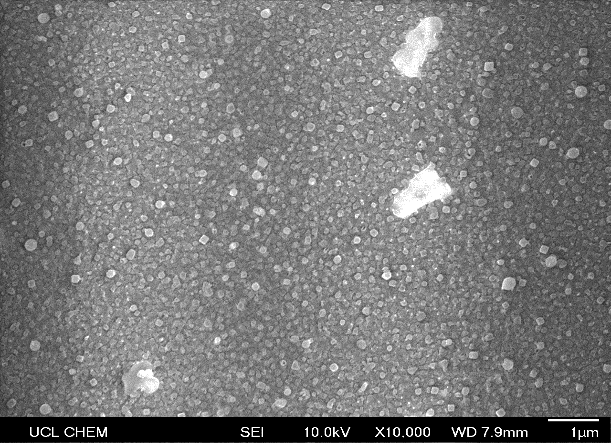

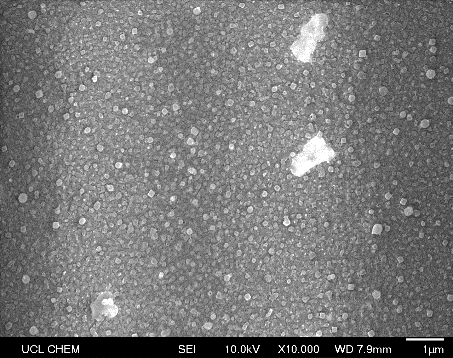

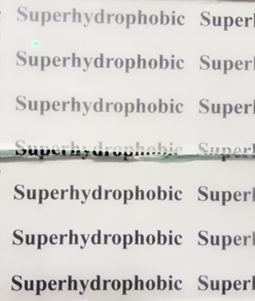


b)

**Fig. S2** Visual comparison of transparency and SEM images between TEOS layer deposited at 450°C for 5 min a) and MPS layers at 450° for 5 min b).


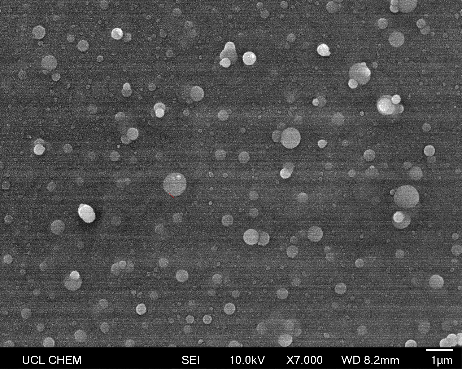


b)

0.365µm

0.750µm

0.591µm

0.419µm

a)


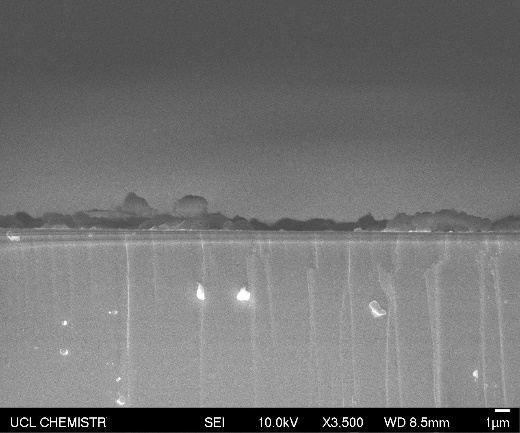


**2.170µm**

**1.473µm**

**Fig. S3** The cross-section a) and top down b) SEM imagines. In a) thickness of MPS-TEOS-POTS film and in b) silica particles size

.

b)

**
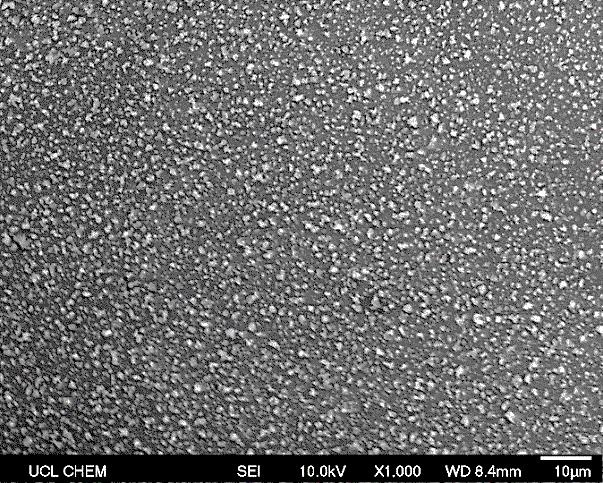

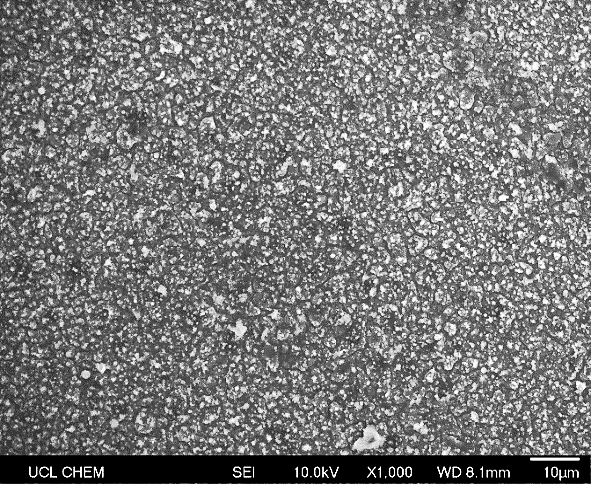
**

a)

**Fig. S4** SEM images of MPS-TEOS-POTS at 5 min a) and 10 min b) of depositions


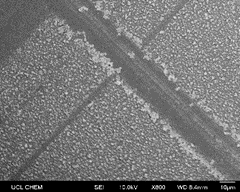

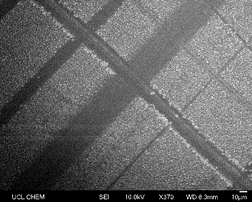


**Fig. S5** SEM images sandpaper test after 20 cycles of abrasion.
